# Exploring Sample Storage Conditions for the Mass Spectrometric Analysis of Extracted Lipids from Latent Fingerprints

**DOI:** 10.3390/biom15040477

**Published:** 2025-03-25

**Authors:** Aleesa E. Chua, Eden P. Go, Heather Desaire

**Affiliations:** Department of Chemistry, University of Kansas, Lawrence, KS 66045, USA; aleesa@ku.edu (A.E.C.); edenp@ku.edu (E.P.G.)

**Keywords:** mass spectrometry, sample processing, storage, lipids, data analysis, variability

## Abstract

In large-scale studies, uncontrolled systematic variability introduced during sample preparation, processing, and storage can interfere with the detection of subtle biological signals. This study evaluates storage conditions, including two sample preparation methods and storage durations, to minimize systematic variability in the analysis of extracted lipids from latent fingerprints. In the traditional approach, samples are prepared immediately, stored as lipid extracts, and processed in multiple batches. In an alternative method, samples are stored directly on the deposition foil, and preparation is delayed until all can be processed in a single batch. Storage duration is evaluated to determine if shorter storage with analysis in multiple batches is more effective than longer storage with analysis in a single batch. Our findings demonstrate that storage of latent fingerprint samples on the deposition foil is a viable option, with minimal degradation of key features even after eight months of storage. While some differences in lipid profiles were observed across storage conditions, these differences were minor and would likely have little impact in larger studies where biological variability is greater. These insights offer practical guidance for implementing latent fingerprint sampling in large-scale studies by identifying optimal conditions that preserve sample quality and streamline workflows.

## 1. Introduction

Lipids play a key role in inflammation [1] and disease progression [2,3], and as such, lipid analysis can facilitate a greater understanding of the mechanisms that underlie health and disease. Consequently, the field of lipidomics is flourishing. Significant advancements in the analysis of lipids have been achieved using commonly leveraged sample sources such as blood and tissue. While these analyses have enabled a better understanding of lipid dysregulation in conditions like obesity and diabetes [4,5] and uncovered biomarkers for various diseases [6,7], the invasive nature of these sample collection methods has limited progress in lipid research surrounding protected populations such as children [8,9] and the elderly [10,11]. As a result, there is growing interest in noninvasive alternative methods that are better suited for these populations.

One promising noninvasive approach involves the analysis of sebum, a lipid-rich substance that can be collected from the skin’s surface [12,13,14]. While previous studies have used slightly less invasive methods, such as the application of a stripping tape to collect sebum [15,16], recent advancements have demonstrated the ability to probe lipid profiles in an even simpler and less invasive way, by transferring skin-surface lipids collected from a subject’s face onto foil surfaces after subjects swipe their fingertips across their face [17,18]. Originally, the analysis of lipids deposited by fingerprints onto various surfaces was a method developed for forensic purposes [19], specifically to determine the age of fingerprints at crime scenes [20,21]. However, a study by Isom et al. has recently adapted this approach to promote noninvasive fingerprint sampling for biomedical applications [17]. In this study, they introduced a noninvasive and high-throughput method for analyzing lipid profiles in groomed latent fingerprints. The method successfully distinguishes between fingerprints collected from different individuals as well as those from specific anatomical regions, such as the cheek, neck, and forehead [17]. This method enables the mass spectrometric (MS) analysis of triacylglycerol and wax ester lipid profiles. Various diseases, such as neurodegenerative disease [22,23,24], cardiovascular disease [25,26], and autism [27,28], have been linked to these lipid classes, highlighting the potential of this method for clinical applications. Furthermore, as sample collection does not involve invasive procedures, this approach is ideal for the inclusion of protected populations, who may otherwise be excluded from studies requiring more invasive sampling methods.

As this method is scaled up for broader clinical applications, it is essential to account for the systematic variability that may be introduced during the sample collection, storage, and analysis processes. Large-scale studies often involve the collection of numerous samples, which, due to the throughput limitations of the analytical instruments and the sample handling capacity of the researchers, typically necessitates batch processing and analysis. The handling of samples in batches can thus introduce variability between batches [29]. If these sources of non-biological variability are not adequately addressed, they can obscure the relevant biological signals, thereby negatively impacting the accuracy and reliability of the results [30,31]. To address this challenge, this study aims to investigate different sample storage conditions to identify the conditions that most minimize non-biological variability.

Specifically, this study investigates how sample preparation and the duration of storage influence the analysis of extracted lipids from latent fingerprints. First, we examine the effects of sample preparation by comparing two approaches: the traditional method of storing samples after sample processing (in their extraction solvent) and an alternative method where fingerprint samples are preserved directly on the deposition foil and prepared later. Preserving samples on foil allows sample preparation to be delayed, enabling all samples to be processed in a single batch. This approach can potentially reduce batch-to-batch variability caused by differences in extraction efficiencies or sample handling across multiple preparation steps. Additionally, this study evaluates the impact of storage duration by examining whether it is more effective to store samples for a short period and analyze them in several smaller batches or to store them for a longer period and analyze them in one large batch. Since longer storage durations can lead to varying degrees of lipid degradation, it is important to understand the impact of storage duration on the lipid profile. By investigating these two variables, this study aims to identify the best conditions that minimize the introduction of unwanted systematic variability while optimizing convenience for the researchers. These insights will be valuable in making latent fingerprint sampling more amenable for large-scale studies and driving future advancements in the field.

## 2. Materials and Methods

### 2.1. Fingerprint Collection

The following experiments were performed in accordance with approved protocols by the University of Kansas’s Human Research Protection Program. Fingerprint samples were collected from two female donors, who were between 20 and 25 years old. In one collection period, four sets of eight fingerprints were collected from each donor, with sets collected at least an hour apart. Over the course of eight months, sixteen collection periods took place, resulting in a total of 1024 samples.

Prior to fingerprint collection, participants were instructed to clean their hands by wiping with a Purell Hand Sanitizing Wipe and allowing them to air dry. Then, they rubbed their hands together for ten seconds to allow for the even distribution of skin surface residues. Afterward, participants followed a ‘grooming’ procedure, where four fingers (excluding thumbs) from each hand were swiped in a back-and-forth motion on the forehead for approximately ten seconds. Each hand was placed on the respective side of the forehead, with the left hand rubbing the left side and the right hand rubbing the right side. Following the grooming process, the participants deposited all eight fingerprints onto eight precut aluminum foil pieces, measuring 1 cm × 2 cm, by applying moderate pressure for ten seconds. These samples were randomly split into two groups to introduce the variable of ambient oxidation [20]. Samples were either processed and/or stored immediately, forming the ‘fresh set of samples, or allowed to oxidize for 24 h, forming the ‘oxidized’ set of samples. Within each group, samples were further separated into one of four subgroups: short-term extract, long-term extract, short-term foil, and long-term foil, where the “foil” groups underwent no sample preparation prior to storage. The preparation of samples within each subgroup is detailed in the following sections. An overview of the sample collection is provided in the Supporting Information.

### 2.2. Sample Preparation

Fingerprint samples were prepared by adapting a method previously described by Isom et al. [17]. Briefly, the foil pieces were folded in half using clean tweezers and placed in a glass vial with 400 µL of dichloromethane. The samples were then sonicated for ten minutes. After sonication, the foil pieces were removed, and 200 µL of water was added to the vial to desalt the samples by liquid–liquid extraction. The mixture was then sonicated for another ten minutes. Samples were allowed to sit for thirty minutes to allow for phase separation. The upper aqueous layer was removed, and the lipid extract was stored in the freezer at −20 °C until analysis. Prior to MS analysis, samples were diluted 20× with methanol and dichloromethane (4:1).

Samples designated as ‘extract’ samples were prepared exactly as above, immediately upon sample collection. Samples designated as ‘foil’ samples were stored in the freezer by placing the foil in the glass vial without the dichloromethane and prepared as above closer to the date of MS analysis. Samples designated as ‘Short-Term’ were stored for a period of zero to three months prior to MS analysis, while samples designated as ‘Long-Term’ were stored for periods spanning zero to eight months. Further details on storage durations are included in Table S1 of the Supporting Information.

### 2.3. Flow Injection Mass Spectrometry

Fingerprint samples were analyzed by high-resolution direct flow injection on an Orbitrap Fusion Tribrid mass spectrometer (Thermo Scientific, San Jose, CA, USA) coupled to a Waters Acquity UPLC (Milford, MA, USA). Samples stored for a short duration were analyzed in three small batches over the eight-month study period, while the samples stored for a longer duration were analyzed in a single larger batch upon completion of sample collection, eight months after the first samples were collected. Samples were injected in a random order. Five microliters of each sample were directly infused from the WATERS Acquity UPLC autosampler to the mass spectrometer using a capillary tubing (50 µm i.d., 650 mm in length) at a flow rate of 25 µL/min. An isocratic elution was employed, with 50% of mobile phase A for two minutes. Mobile phase A consisted of methanol/water (50:50) containing 5 mM ammonium acetate, while mobile phase B consisted of acetone/2-propanol/water (20:79:1) containing 5 mM ammonium acetate. A short wash and a blank run were performed between every sample to ensure no sample carryover.

Mass spectrometric analysis was performed in the positive ion mode using the following parameters: a spray voltage of 2.9 kV, sheath gas: five units, auxiliary gas: seven units, the ion transfer tube maintained at 275 °C, and the vaporizer set to 45 °C. Full MS scans in the Orbitrap were performed in 3 s cycles over a mass range of 250–1400 *m*/*z* at a resolution of 120,000 at 200 *m*/*z*. The AGC target value was set to 4 × 10^5^ with a maximum injection time of 50 ms.

### 2.4. Data Analysis

The raw MS data (.RAW files) were first converted to the .MS1 format using RawConverter (Scripps, Version 1.2.0.0) [32]. Then, an in-house R script, provided in the Supporting Information of reference [17], was used to read in the MS1 files and generate the feature matrices used for supervised and unsupervised classification.

To create the feature matrices, the script processed signal intensities within a 0.05-min range, where the exact retention time was optimized for each batch of samples to capture the maximum signal. The mass range of 250–1400 *m*/*z* was divided into 0.1 Da bins. Each feature in the resulting matrix corresponds to the total ion intensity for a given bin, obtained by summing all intensities that fall in that 0.1 Da bin for each sample over the 0.05 min range. Further details on the binning process can be found in reference [18]. To reduce noise, each matrix was filtered so that only bins with a nonzero value for at least 1% of all samples were included. This filtering step results in a total of 11,167 features for each matrix. Quantile normalization was then applied to each matrix using the limma package from Bioconductor.

The resulting matrices were first transposed and then analyzed by principal components analysis (PCA) and supervised classification using XGBoost (R package “xgboost”) and AC.2021 [33,34]. Visualization by PCA was performed using the R package “factoextra.” Supervised classification was conducted to discriminate samples based on donor and ambient oxidation. For XGBoost classification, the following hyperparameters were used: objective = “binary:logistic”, eta = 0.3, gamma = 0, max_depth = 6, min_child_weight = 1, subsample = 1, colsample_bytree = 1. For AC.2021 classification, X was set to 8 and Repeats was set to 1000. Leave-one-group-out cross-validation (LOGOCV) was used with both classifiers, where all samples from the same donor, prepared and stored under identical conditions and collected during the same period, were excluded from each validation step. Sample code for LOGOCV in AC.2021 is provided in the Supporting Information. To evaluate supervised classification performance, the classification accuracies and area under the receiver operating characteristic curve (AUC) were calculated. The AUC values and statistical significance calculations via the DeLong test were performed using the R package pROC.

## 3. Results and Discussion

**Figure 1:**
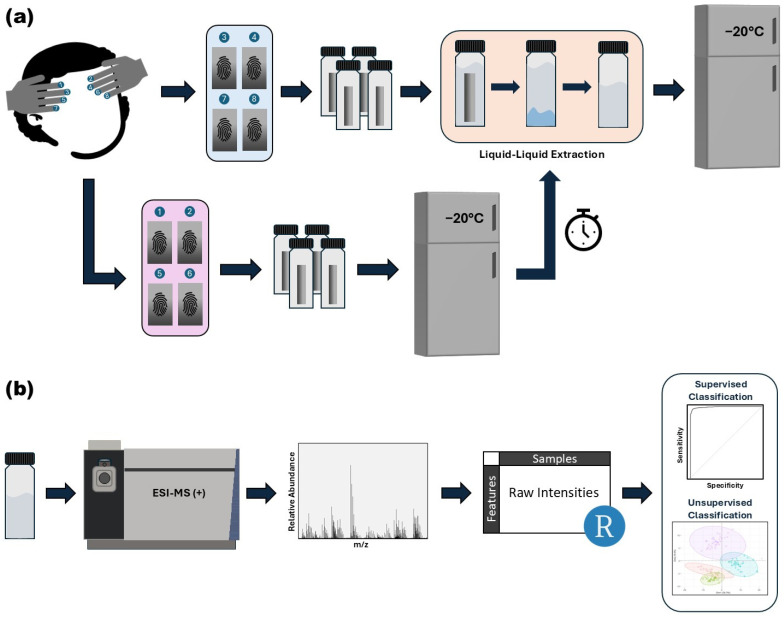
Workflow for sample collection, preparation, data acquisition, and analysis. (**a**) Latent fingerprints are first groomed on the forehead and deposited onto precut foil pieces. Eight fingerprints are collected in each collection, which are randomly divided into two groups. One group (blue) is processed immediately and stored in the extraction solvent, while the other group (purple) is stored on the deposition foil and processed closer to the time of analysis. (**b**) Samples are analyzed using high-resolution flow injection mass spectrometry with electrospray ionization in the positive ion mode. The resulting mass spectra are binned, converted into a matrix, and normalized in R. The processed data are then used for subsequent analysis employing supervised and unsupervised classification techniques.

This paper aims to identify conditions that best minimize systematic variability in the analysis of triacylglycerols and wax esters from latent fingerprints by investigating the effects of sample preparation and storage duration. In this study, samples were either prepared immediately after collection and stored in their extraction solvent or stored on the deposition foil and processed closer to the time of analysis. To assess the effects of storage duration, samples prepared under both methods were collected and stored for either a short period of zero to three months, or a longer period, extending up to eight months, prior to analysis by mass spectrometry. To mimic a biomarker study, where the variable of interest might be distinguishing healthy and diseased samples, we introduced two different variables of interest, by first collecting samples from two donors and also by processing samples at two different time points after deposition (0 h, 24 h). These time points reflect the effects of ambient oxidation, and they offer two distinct sample types that have previously been shown to exhibit differences in lipid profiles [20,35]. This experimental design enables the evaluation of the effects of various storage conditions in the context of a discrimination task (where either the individual is discriminated or the presence of ambient oxidation is assessed). For clarity, an overview of the variables and their reason for inclusion is provided in Table 1. The resulting MS data were then analyzed by different supervised and unsupervised classification tools. The workflow for sample collection, processing, and data acquisition is shown in Figure 1.

### 3.1. Test 1: Unsupervised Classification

In large studies where biological differences may be subtle, systematic variability from sample storage and processing can often overshadow relevant biological signals. One way to determine if this variability is present is through a principal components analysis (PCA). As PCA clusters samples based on the source of greatest variability, this analysis can quickly reveal whether specific storage conditions impart the greatest differences in lipid profiles, or if other factors are more influential.

**Figure 2:**
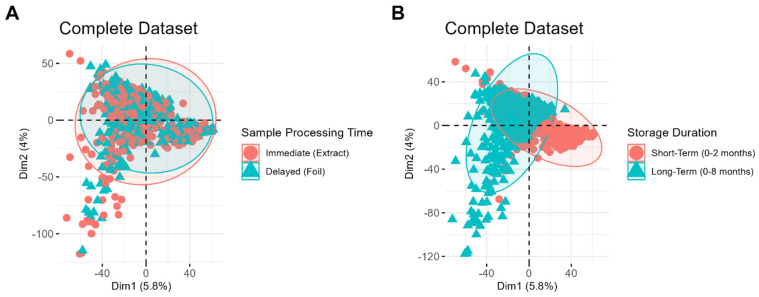
Principal components analysis (PCA) score plots for method variable differentiation. Sample points on the PCA shown in (**A**) correspond with samples processed immediately (red circle) vs. samples processed later (blue triangle), while those on panel (**B**) correspond with samples stored for a short period (red circle) and those stored for a longer period (blue triangle).

The resulting PCA score plots are shown in Figure 2. Figure 2A,B depict the same set of samples, labeled based on different variables, to demonstrate which clustering patterns are most evident in the dataset. No distinct clustering is observed between samples processed immediately and those stored first and processed later (Figure 2A), suggesting that the timing of sample processing is a relatively minor source of variability compared to other factors. In contrast, distinct clustering is evident between samples of different storage durations (Figure 2B), highlighting storage duration as a significant contributor to systematic variability in the dataset. Interestingly, tighter clustering is observed among samples stored for a short period, whereas samples stored for longer durations display a greater spread. This difference highlights the increased variability among long-term samples, which may be attributed to the wider range of storage times and potential chemical changes, such as lipid degradation, that occur over prolonged storage durations.

**Figure 3:**
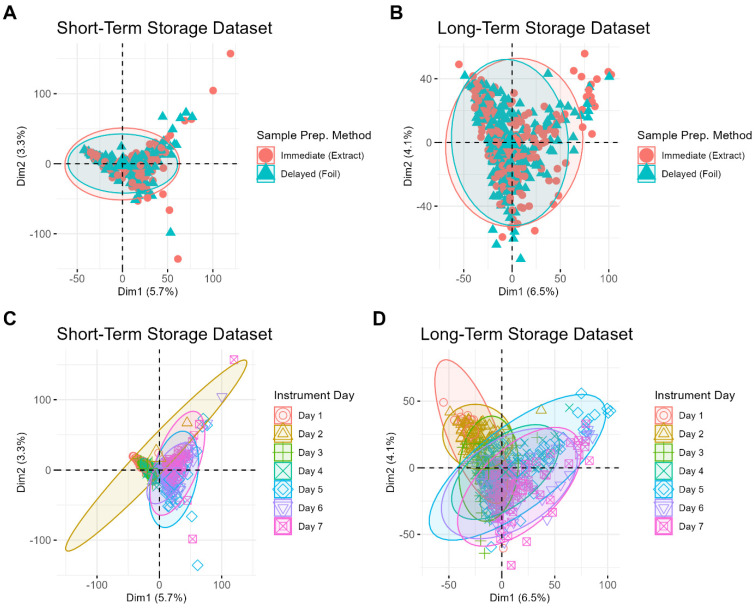
Principal components analysis (PCA) score plots for short-term storage (**A**,**B**) and long-term storage (**C**,**D**) samples. Sample points on the PCA shown in (**A**,**B**) correspond with samples processed immediately (red circle) vs. samples processed later (blue triangle), while those on panels (**C**,**D**) correspond to different days of MS data acquisition.

To further explore these findings, storage duration was removed as a variable, and short-term and long-term storage samples were analyzed separately (Figure 3). Even with storage variability removed, samples do not cluster based on sample preparation method (Figure 3A,B). Instead, clustering is observed based on the date of MS data acquisition for both the short-term storage (Figure 3C) and long-term storage (Figure 3D) datasets. This highlights day-to-day instrument variability as another contributor to the overall variability in the datasets.

**Figure 4:**
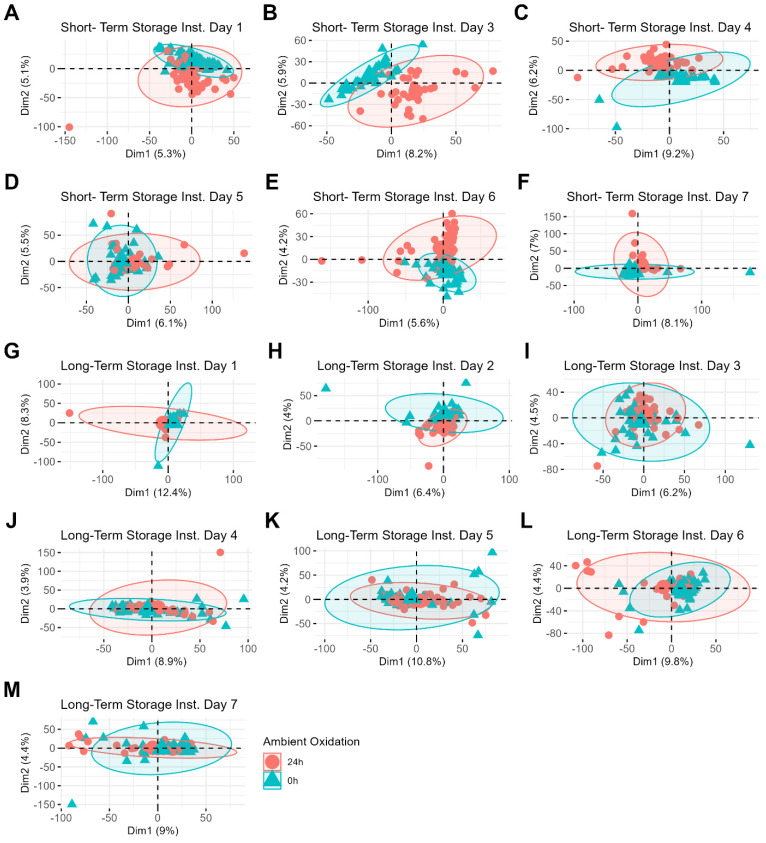
Principal components analysis (PCA) for ambient oxidation differentiation, by instrument day. Sample points on the PCA correspond with samples stored for a short period (**A**–**F**) and those stored for a longer period (**G**–**M**). Instrument day two was excluded due to the small number of samples ran on that day.

Even when performing PCA for each batch of samples separately, the data still do not cluster based on the sample preparation method, further demonstrating that this variable does not significantly impact the data (Supporting Information Figure S2). This reinforces the finding that the sequence of storage and preparation contributes minimally to the overall variability in the dataset and suggests that both immediate and delayed processing methods are equally viable. Instead, sample differences related to the classification variables (donor and ambient oxidation) become more apparent, particularly under the variable of ambient oxidation, which is shown in Figure 4. For example, a PCA plot of short-term storage samples whose MS data were acquired on day three shows clear clustering between freshly prepared samples and those prepared 24 h after deposition (Figure 4B). Interestingly, the clustering patterns related to sample aging become less distinct for samples stored over longer periods (Figure 4H–L). The increased difficulty in identifying these clustering patterns may be attributed to the additional variability introduced by the wider range of storage times for these samples (0 to 8 months compared to 0 to 3 months). Donor differences, on the other hand, are less easily identifiable by PCA, even when the samples are restricted to individual batches (Supporting Information Figure S3), which suggests that these differences are more subtle than those driven by the sample oxidation process. Notably, the relative overall variation is small, as evidenced by the low percentage of variance captured by the first two principal components (Figure 2, Figure 3 and Figure 4). Altogether, these findings highlight that batch variability in MS data acquisition is the primary source of variation in the dataset, rather than the sample preparation method or storage duration.

### 3.2. Test 2: Supervised Classification

**Figure 5:**
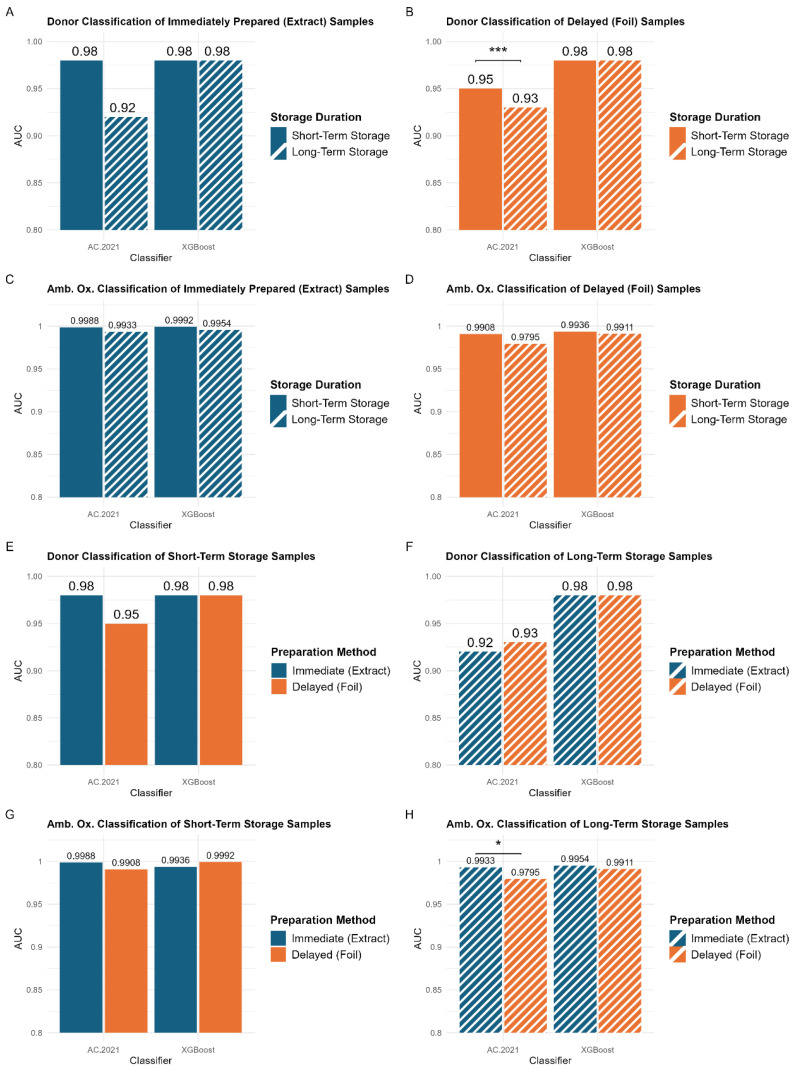
Area under the curve (AUC) values for donor and ambient oxidation classification. Panels (**A**–**D**) compare the model performance for samples stored for short (solid bars) and extended (dashed bars) durations using the AC.2021 and XGBoost models, while Panels (**E**–**H**) compare the model performance between the immediate (blue bars) and delayed (orange bars) preparation methods. Statistical significance is marked as *** (*p* < 0.005) and * (*p* < 0.05).

Optimal storage conditions are expected to introduce less systematic variability which leads to improved supervised classification outcomes. To evaluate storage conditions, supervised classification was performed using two different methods: XGBoost [33] and AC.2021 [34]. The resulting AUC values for both classifiers across the different data types are summarized in Figure 5. Statistical comparisons between the AUC values are also illustrated in Figure 5, with additional details provided in Tables S2 and S3. Overall, classification outcomes in this study align with prior research, which has demonstrated strong discrimination between samples from different donors [17] and between samples with varying levels of ambient oxidation [18,35].

No significant differences were observed between storage durations or sample preparation methods across all XGBoost classifications (Figure 5A–H). This is likely due to XGBoost’s decision-making process, which relies on a smaller set (~51) of highly informative features. By focusing on the most predictive features, XGBoost may be less affected by variations in storage duration. In contrast, AC.2021 utilizes a larger number (~232) of features, enabling it to capture more subtle differences in classification outcomes across storage durations. While this broader sensitivity provides a more detailed assessment of storage effects, it also makes the model more susceptible to systematic variability, which can reduce overall classification performance. By leveraging a smaller feature set, XGBoost may be more resistant to differences in storage duration, while AC.2021’s more comprehensive approach provides insight into how storage conditions can influence classification outcomes. Given these differences, the following analysis will focus on AC.2021 results to further explore the impact of storage conditions on classification performance.

### 3.3. Comparing Storage Durations

Supervised classification demonstrates that extended storage results in lower AUC values, regardless of the classification problem (Figure 5A–D), although some of the changes were quite small. A statistically significant difference (*p* < 0.005) was observed in the AC.2021 donor classification for samples prepared using the delayed method (Figure 5B, left panel). The model performed better for samples stored for a period of three months or less (AUC = 0.95) than for those stored for a longer period of eight months (AUC = 0.93). These findings indicate that shorter storage durations lead to somewhat better classification accuracy, especially for samples prepared using the delayed method.

To further investigate the impact of storage duration, the percent relative standard deviation for the top features in donor classification was measured for the two sets of samples, those stored for three months or less and those stored for up to eight months. (Tables S4 and S5). These data reveal detectable but statistically insignificant differences between the two sample sets, for both XGBoost and AC.2021 models. While extended storage is associated with a slight reduction in classification performance, the overall variability in these key features remains low, and the RSDs for key features do not become significantly worse with longer storage duration. This suggests that even after eight months, degradation of these key features is minimal, and the impact of storage duration is negligible.

### 3.4. Comparing Sample Preparation Methods

Figure 5E–H compare the classification performance of models trained on samples prepared using the immediate (blue bars) and delayed (orange bars) methods. Unlike the storage duration comparisons (Figure 5A–D), no consistent trend is observed between preparation methods. For short-term storage, no statistically significant differences exist between the two preparation methods (Figure 5E,G). This aligns with the PCA results, indicating that the preparation method does not introduce substantial variability. Thus, for storage durations of three months or less, samples can be stored either in their extraction solvents or preserved on the deposition foil, without affecting classification performance. This flexibility is particularly beneficial in clinical settings where immediate processing may not always be possible. However, a small but statistically significant difference is observed in the classification of the ambient oxidation variable for long-term storage samples (Figure 5H). Models trained on samples prepared using the immediate method (AUC = 0.9933) performed significantly better than those trained on samples prepared using the delayed method (AUC = 0.9795). This suggests that for extended storage durations (>3 months), the optimal approach would involve preparing samples immediately. However, it is important to note that these differences remain very subtle and would likely become negligible with the introduction of greater biological variability in larger studies.

Ultimately, these results emphasize the flexibility available to researchers in the methods they choose for latent fingerprint samples. For both sample preparation methods, storage for shorter durations (within three months) is ideal, but longer storage periods did not degrade the results very much. One of the two classification tools (XGBoost) did not detect a drop in classification accuracy, but the other did detect some drop-off. Longer storage can increase the likelihood of lipid degradation, which is likely the primary factor contributing to the slightly lower performance of samples stored for longer periods. This finding is further supported by the less distinct clustering observed in the principal components analysis of the sample aging variable for samples stored over longer periods (Figure 4).

## 4. Conclusions

This study offers key insights into optimizing noninvasive latent fingerprint sampling methods for large-scale and clinical lipidomics research. We evaluated sample storage conditions to minimize non-biological variability, particularly variability introduced during batch processing. Our results show that storage of samples on foil is an effective alternative to processing the samples at the time of collection, making sample collection more practical in clinical settings, particularly for periods of three months or less. Additionally, we found that storing samples prior to MS analysis within up to eight months showed very little impact overall on the lipid profiles. These findings offer practical guidance for future large-scale studies that utilize noninvasive fingerprint sampling methods. With these sample storage considerations in mind, these methods become better suited for clinical settings and can contribute to the greater inclusivity of sample collection, particularly for populations that might otherwise be excluded due to invasive procedures.

## Figures and Tables

**Table 1:** An overview of classification and method variables used in this study.

**Category**	**Variable**	**Reason for Inclusion**
Classification Variables	Donor	Lipids have observable biological variability; used to evaluate whether method variables influence the ability to detect these differences.
	Ambient Oxidation	Lipids oxidize to a modest extent in 24 h at room temperature; used to evaluate whether method variables influence the ability to detect these differences.
Method Variables	Storage Duration	To evaluate whether short-term storage (0–3 months) vs. long-term storage (0–8 months) affects the ability to classify samples based on known differences.
	Sample Preparation	To determine whether immediate or delayed processing affects the ability to classify samples based on known differences.

## Data Availability

The raw data for this study can be found in Table S6 of the Supplementary Materials.

## Supplementary Materials

The following supporting information can be downloaded at https://www.mdpi.com/article/10.3390/biom15040477/s1: Method S1: Code for leave-one-group-out classification using AC.2021; Figure S1: Overview of all samples collected for the study; Figure S2: Principal components analysis score plots for sample processing time differentiation; Figure S3: Principal components analysis score plots for donor differentiation; Table S1: Sample collection periods and their corresponding storage durations; Table S2: Supervised classification results for samples stored for a period of three months or less; Table S3: Supervised classification results for samples stored for a period extending up to eight months; Table S4: Median percent relative standard deviation for the top features in donor classification by XGBoost; Table S5: Median percent relative standard deviation for the top features in donor classification by AC.2021; Table S6: Raw feature matrices.
