# Supplementary material for: Exploring Sample Storage Conditions for the Mass Spectrometric Analysis of Extracted Lipids from Latent Fingerprints
Source: Biomolecules. 2025 Mar 25;15(4):477. doi: 10.3390/biom15040477 (PMC12024923; doi:10.3390/biom15040477)
Supplement: Supplementary file 1 [file biomolecules-15-00477-s001.zip › FPStorage_SI_v2.pdf]

**Supporting Information for:**

# Exploring Sample Storage Conditions for the Mass Spectrometric Analysis of Extracted Lipids from Latent Fingerprints


Aleesa E Chua[1], Eden P Go[1], Heather Desaire[1*]

[1] Department of Chemistry, University of Kansas, Lawrence, Kansas, United States, 66045

*Address correspondence to: Heather Desaire, phone: 785-864-3015, Email: hdesaire@ku.edu


**Table of Contents:**


| | |
|---|---|
| Method S1 | 2 |
| Figure S1 | 4 |
| Figure S2 | 5 |
| Figure S3 | 6 |
| Table S1 | 7 |
| Table S2 | 7 |
| Table S3 | 8 |
| Table S4 | 8 |
| Table S5 | 8 |
| Table S6 | (xlsx file) |

**Method S1.** Code for Leave One Group Out Classification Using AC.2021

```
 {
  final_df_mat <- as.matrix(input_matrix)
  AC_df <- rbind(final_df_mat, 1)
  feature_matrix <- AC_df[,1:ncol(input_matrix)]
  {
    features <- as.numeric(nrow(feature_matrix))
    samples <- as.numeric(ncol(feature_matrix))
    Low<-c(which(your_key==0))
    Hi<-c(which(your_key==1))

    r<-features
    C<-samples
    InputMat<-as.matrix(feature_matrix)
    X<- 8
    Repeats <- 1000
    set1<-c(1:X)
    set2<-c(1:X)
    Output2Mat<-matrix(0,r,C)
    FeatureCount<-matrix(0,r,C)
    ThirtyByEightMatrix<-matrix(0,r,(2*X))
    RatioVec<-c(1:r)
    SplitVec<-c(1:r)
    NewVec<-1:r
    Results<-c(1:C)
    Results[ ]<-0
    AnsVec<-c(1:C)
    AnsVec[ ]<-0

    uniquekeys = unique(your_LOOkey)

    for (m in (uniquekeys)) {

      for (k in 1:Repeats)

      { Low_LOOkey<-your_LOOkey[which(your_key==0)]
        Hi_LOOkey<-your_LOOkey[which(your_key==1)]

        set1<-sample(Low[-which(Low_LOOkey%in%m)], X, replace=F)
        set2<-sample(Hi[-which(Hi_LOOkey%in%m)], X, replace=F)
        RatioVec[]<-0
        SplitVec[]<-0
        Output2Mat[ , ]<-0
        ThirtyByEightMatrix<- InputMat[ , c(set1,set2)]
        ;
        #The module below selects the features to keep.
        for (i in 1:r)
          (ifelse (xor(min(ThirtyByEightMatrix [i,1:X])> max(ThirtyByEightMatrix[i,(X+1):(2*X)]),
max(ThirtyByEightMatrix [i,1:X])<min(ThirtyByEightMatrix[i,(X+1):(2*X)])),
              RatioVec[i]<-1, RatioVec[i]<-0))
```

```
        ;
        #The module below assigns the border between the two groups.
        for (i in 1:r)
          (
           ifelse (RatioVec[i]==1,
                  SplitVec[i]<-
(ifelse(sum(ThirtyByEightMatrix[i,1:X])>sum(ThirtyByEightMatrix[i,(X+1):(2*X)]),

                                    (min(ThirtyByEightMatrix[i,1:X])+
max(ThirtyByEightMatrix[i,(X+1):(2*X)]))/2,
                                    (min(ThirtyByEightMatrix[i,(X+1):(2*X)])+
max(ThirtyByEightMatrix[i,1:X]))/2)),
                  SplitVec[i]<-0)
          )
        ;
        for (i in 1:r)
          (
           NewVec[i]<-as.numeric(ThirtyByEightMatrix[i,1]>SplitVec[i])
          )
        NewVec[which(NewVec==0)]<- (-1)
        for (i in setdiff(1:C,c(set1,set2)))
          (
           Output2Mat[,i]<-as.numeric(InputMat[,i]>SplitVec)
          )
        Output2Mat[which(Output2Mat==0)]<-(-1)

        for (i in setdiff(1:C,c(set1,set2)))
          (Output2Mat[ ,i]<-Output2Mat[ ,i]* (NewVec*RatioVec))
        ;
        Output2Mat[,c(set1,set2)]<-0
        Output2Mat[is.na(Output2Mat)]<-0

        FeatureCount<-Output2Mat+FeatureCount
        is_divisible_by_100 <- function(k) {
          return(k %% 100 == 0)
        }
}
      }

      for (i in 1:C)
        (Results[i]<-c(sum(FeatureCount[ ,i])))
    }
    group_leftout = which(your_LOOkey%in%m)
    AnsVec[group_leftout] = Results[group_leftout]
    }
```

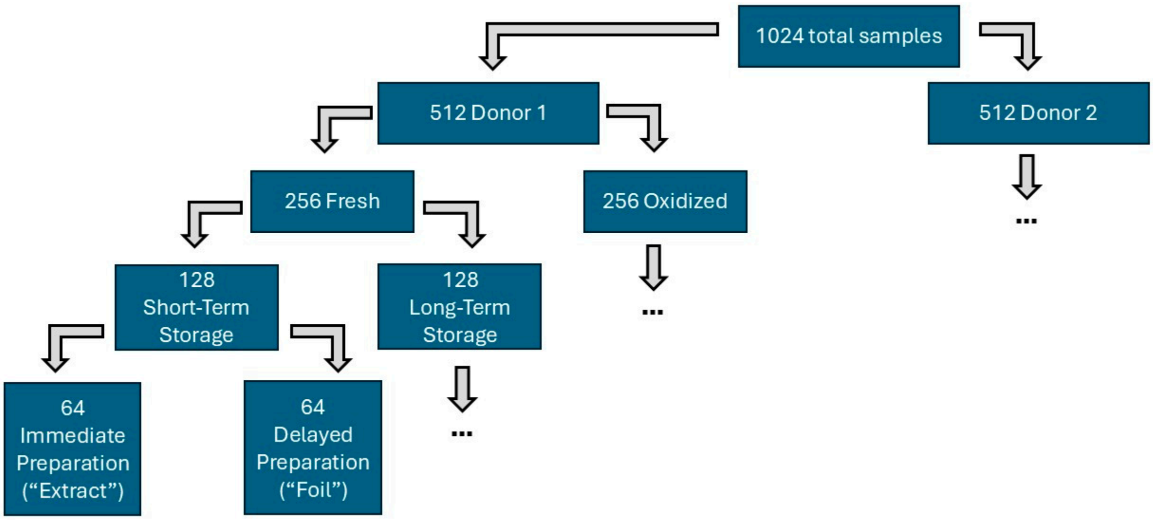


**Figure S1.** Overview of all samples collected for the study. A total of 1024 samples were obtained, with 512 samples per donor (Donor 1 and Donor 2). Each donor's samples were divided into two categories to generate the ambient oxidation variable: half were prepared immediately, forming the "Fresh" set while the other half were exposed to ambient air for 24h to form the "Oxidized" set of samples. These two categories were further split into short-term and long-term storage durations. Each storage group was then divided based on the preparation method, using either the delayed approach (storage on deposition foil prior to preparation) or the immediate approach (storage of the prepared sample as a lipid extract). The "…" in the figure indicates that the same sample breakdown was applied at each subsequent step, as shown to the left.

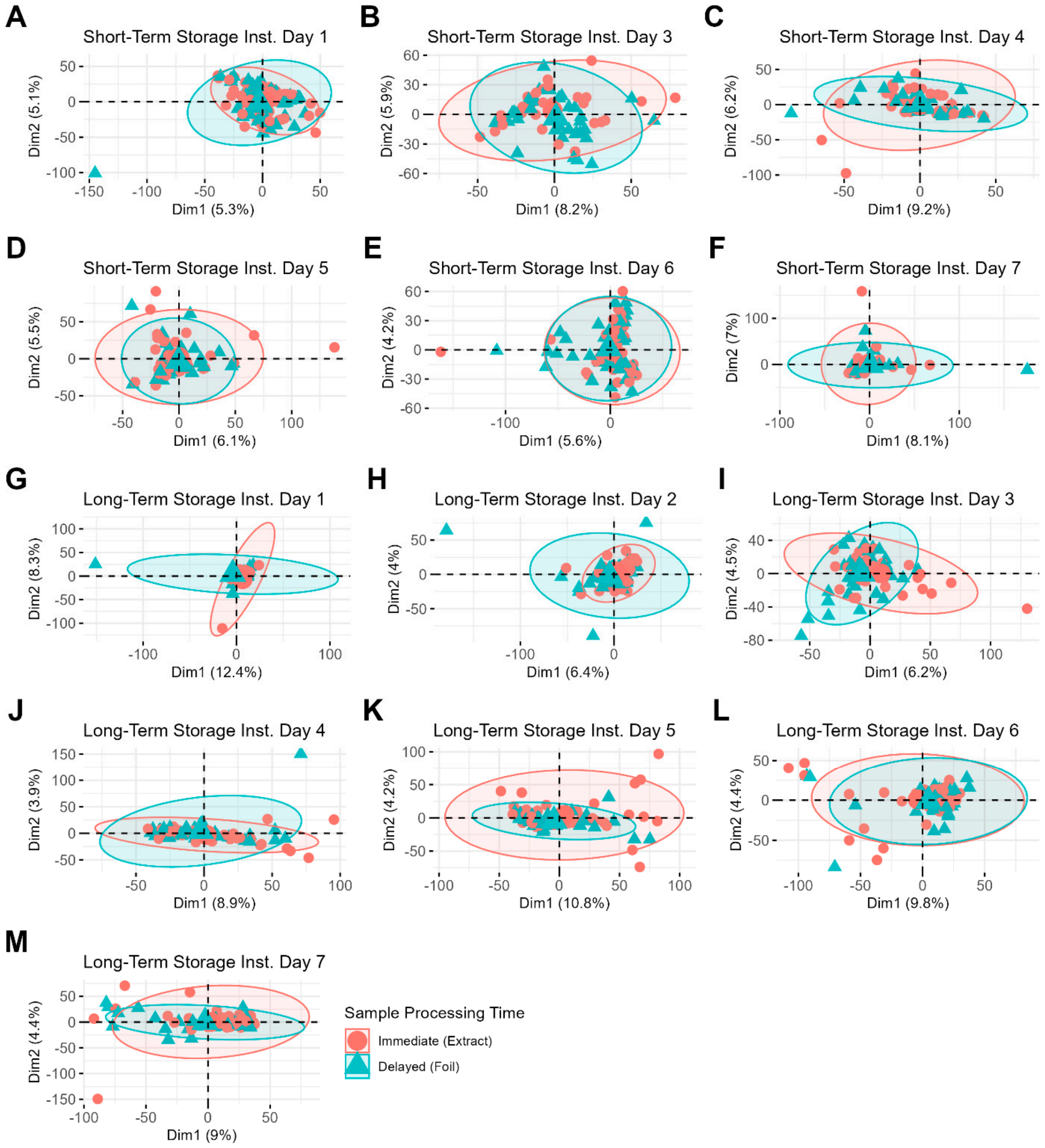


**Figure S2.** Principal components analysis score plots for sample processing time differentiation, with each instrument day assessed separately. Sample points on the PCA correspond with samples stored for a short period (A-F) and those stored for a longer period (G-M). Instrument day two was excluded due to the small number of samples ran on that day.

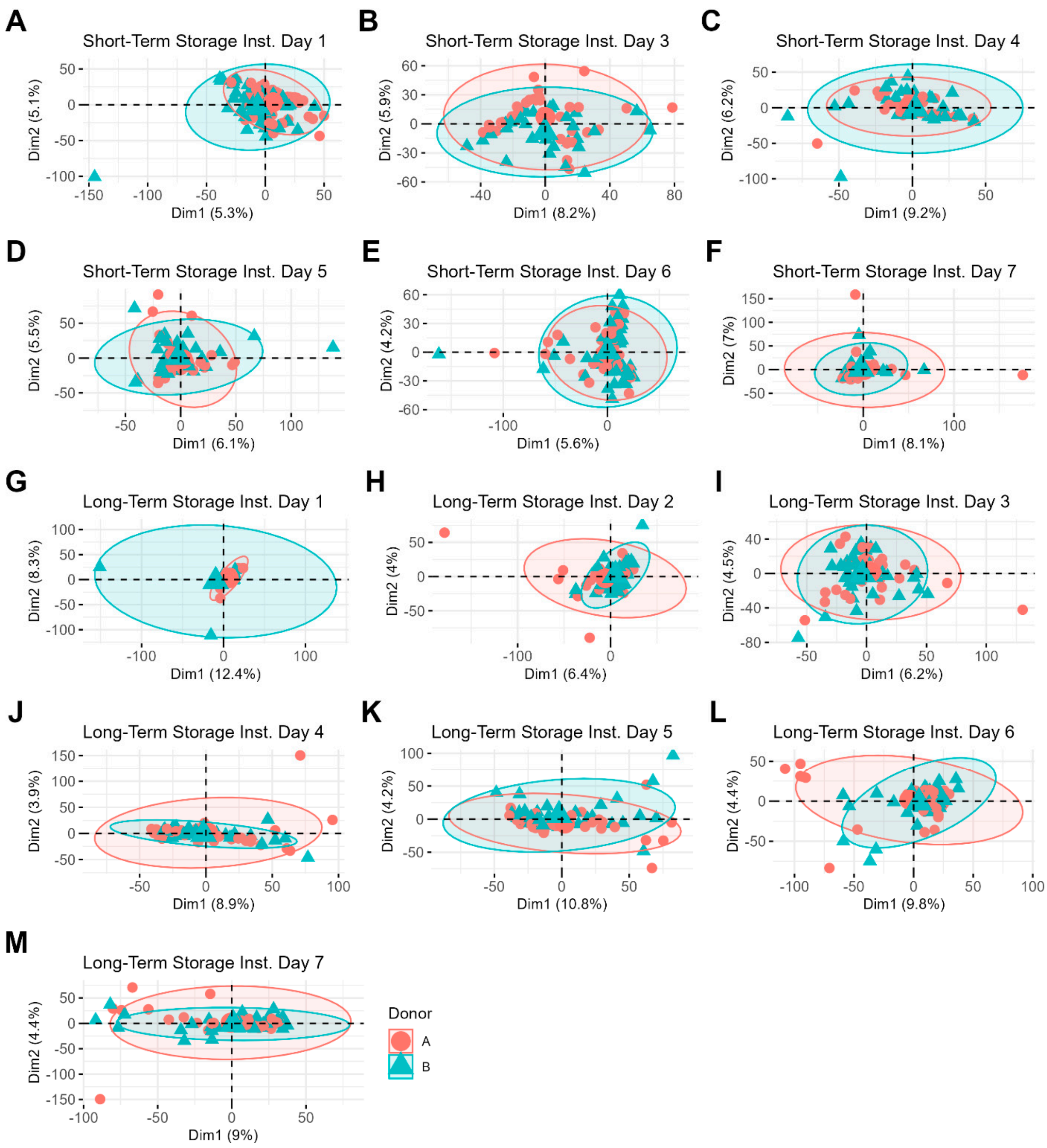


**Figure S3.** Principal components analysis score plots for donor differentiation, with each instrument day assessed separately. Sample points on the PCA correspond with samples stored for a short period (A-F) and those stored for a longer period (G-M). Instrument day two was excluded due to the small number of samples ran on that day.

**Table S1.** Sample collection periods and their corresponding storage durations.

| Collection Period | Storage Duration of "Short-Term" Storage Samples (in months) | Storage Duration of "Long-Term" Storage Samples (in months) |
|---|---|---|
| 1 | 3 | 8 |
| 2 | 3 | 7 |
| 3 | 2 | 6 |
| 4 | 2 | 6 |
| 5 | 1 | 5 |
| 6 | 1 | 5 |
| 7 | 0 | 4 |
| 8 | 2 | 3 |
| 9 | 2 | 3 |
| 10 | 2 | 3 |
| 11 | 1 | 2 |
| 12 | 1 | 2 |
| 13 | 2 | 2 |
| 14 | 1 | 1 |
| 15 | 1 | 1 |
| 16 | 0 | 0 |

**Table S2**. Supervised classification results for samples stored for a period of three months or less.

<table>
<tr><th>Storage Duration</th><th>Classifier</th><th>Classification Problem</th><th>Sample Processing Time</th><th>Accuracy</th><th>AUC</th></tr>
<tr><td rowspan="8">Short (0-3 months)</td><td rowspan="4">AC.2021</td><td rowspan="2">Donor</td><td>Immediate (Extract)</td><td>92%</td><td>0.9782</td></tr>
<tr><td>Delayed (Foil)</td><td>91%</td><td>0.9529</td></tr>
<tr><td rowspan="2">Ambient Oxidation</td><td>Immediate (Extract)</td><td>97%</td><td>0.9988</td></tr>
<tr><td>Delayed (Foil)</td><td>96%</td><td>0.9908</td></tr>
<tr><td rowspan="4">XGBoost</td><td rowspan="2">Donor</td><td>Immediate (Extract)</td><td>92%</td><td>0.9750</td></tr>
<tr><td>Delayed (Foil)</td><td>94%</td><td>0.9783</td></tr>
<tr><td rowspan="2">Ambient Oxidation</td><td>Immediate (Extract)</td><td>100%</td><td>0.9992</td></tr>
<tr><td>Delayed (Foil)</td><td>98%</td><td>0.9936</td></tr>
</table>

**Table S3**. Supervised classification results for samples stored for a period extending up to eight months.

| Storage Duration | Classifier | Classification Problem | Sample Processing Time | Accuracy | AUC |
|---|---|---|---|---|---|
| Long (0-8 months) | AC.2021 | Donor | Immediate (Extract) | 86% | 0.9178 |
| | | | Delayed (Foil) | 87% | 0.9290 |
| | | Ambient Oxidation | Immediate (Extract) | 90% | 0.9933 |
| | | | Delayed (Foil) | 87% | 0.9795 |
| | XGBoost | Donor | Immediate (Extract) | 93% | 0.9844 |
| | | | Delayed (Foil) | 91% | 0.9795 |
| | | Ambient Oxidation | Immediate (Extract) | 97% | 0.9954 |
| | | | Delayed (Foil) | 96% | 0.9911 |

**Table S4**. Median percent relative standard deviation for the top ten* features in donor classification by XGBoost, averaged for the two donors across both oxidized and unoxidized samples.

| Storage Duration | Immediate Processing | Delayed Processing |
|---|---|---|
| Short (0-3 months) | 7% | 8% |
| Long (0-8 months) | 8% | 8% |

* The features used for RSD analysis were selected based on a gain value greater than 0.01 in donor classification.

**Table S5**. Median percent relative standard deviation for the top 84* features in donor classification by AC.2021, averaged for the two donors across both oxidized and unoxidized samples.

| Storage Duration | Immediate Processing | Delayed Processing |
|---|---|---|
| Short (0-3 months) | 9% | 9% |
| Long (0-8 months) | 9% | 10% |

* The features used for RSD analysis were selected if their FeatureScore was at least 0.01% of the maximum FeatureScore in donor classification.
